# Case of Colica Pictonum

**Published:** 1873-07

**Authors:** John P. Ralls

**Affiliations:** Gadsden, Alabama


### CASE OF COLICA PICTONUM.

BY JOHN P. RALLS, M.D., GADSDEN, ALABAMA.

Called to see M. S., a lad eighteen years of age, by occupation a printer. Found him in bed, complexion somewhat pale, pulse but little accelerated, and but little heat of skin. Complains considerably of pain in the stomach and bowels, which, on examination, are found to be not at all tympanitic. The extensor muscles of the forearm and hand are paralyzed to such a degree that the hand is flexed upon the wrist, (wrist drop,) and the fingers are flexed upon the hand. The gums, along the line of their junction with teeth, presented the characteristic bluish, or purplish colored tinge. In a word, all the symptoms indicated a well-developed case of colica pictonum. To relieve the distressing pain, opiates were prescribed internally, and cloths wrung out of hot water were ordered to be applied to the stomach and abdomen. These remedies were followed by an active saline purgative to relieve the constipation. The patient was then put on iodide potassium regularly continued, with opiates occasionally, as he still suffered more or less from abdominal pains. The improvement was gradual, and in about two weeks he was enabled to resume work. The feature of prime interest in this case was the question as to what could have given rise to the attack. We know that painters and those who work in lead are liable to have their constitutions undermined by the absorption of this slow poison, but I do not remember that writers mention the mere handling of type as a source of danger in this respect. I expressed my surprise to the mother of the patient that the mere handling of type should have caused the severe suffering that she was now witnessing in her son, (for I was fully satisfied that there was no other source of the disease in this instance,) as the solid lead only touched the tips of the fingers, and that the skin on these was comparatively dry, considerably thickened and hard, from constant use, and that we might therefore hardly expect the system to be poisoned in this way. To this she replied by stating that her son, from a child, when handling any thing, was accustomed to pass his fingers frequently through his lips and across the tip of his tongue, that, his fingers being moistened, he might the better manipulate whatever he had in hand, adding that she supposed he did the same thing now that he was type-setting. To the latter remark the son at once assented. So here was a revelation of the mystery. It was not the hard, dry fingers that had acted as inlets to disease, but the soft, vascular lips and tongue. We may also suppose that the stomach received its share of the poison in the saliva that was swallowed. I dismissed my patient with an injunction that he must correct the habit as above mentioned, but that if he must needs moisten his fingers to expedite his type-setting, to keep near him a bit of moistened sponge, or other like material that will serve the purpose. This caution, impressed upon him as it has been by days and nights of suffering, he will not likely soon forget.

